# Global analysis of gene expression profiles in the submandibular salivary gland of klotho knockout mice

**DOI:** 10.1002/jcp.26172

**Published:** 2017-09-28

**Authors:** Sung‐Min Kwon, Soo‐A Kim, Jung‐Hoon Yoon, Jong‐In Yook, Sang‐Gun Ahn

**Affiliations:** ^1^ Department of Oral and Maxillofacial Pathology, College of Dentistry Wonkwang University Daejeon Republic of Korea; ^2^ Department of Biochemistry, College of Oriental Medicine Dongguk University Gyeongju Republic of Korea; ^3^ Department of Oral Pathology Yonsei University College of Dentistry Seoul Republic of Korea; ^4^ Department of Pathology, College of Dentistry Chosun University Gwangju Republic of Korea

**Keywords:** aging, gene profiling, klotho, salivary gland

## Abstract

Salivary dysfunction commonly occurs in many older adults and is considered a physiological phenomenon. However, the genetic changes in salivary glands during aging have not been characterized. The present study analyzed the gene expression profile in salivary glands from accelerated aging klotho deficient mice (klotho−/−, 4 weeks old). Microarray analysis showed that 195 genes were differentially expressed (z‐score > 2 in two independent arrays) in klotho null mice compared to wild‐type mice. Importantly, alpha2‐Na^+^/K^+^‐ATPase (Atp1a2), Ca^2+^‐ATPase (Atp2a1), epidermal growth factor (EGF), and nerve growth factor (NGF), which have been suggested to be regulators of submandibular salivary gland function, were significantly decreased. When a network was constructed from the differentially expressed genes, proliferator‐activated receptor‐γ (PPAR γ), which regulates energy homeostasis and insulin sensitivity, was located at the core of the network. In addition, the expression of genes proposed to regulate various PPAR γ‐related cellular pathways, such as Klk1b26, Egfbp2, Cox8b, Gpx3, Fabp3, EGF, and NGFβ, was altered in the submandibular salivary glands of klotho−/− mice. Our results may provide clues for the identification of novel genes involved in salivary gland dysfunction. Further characterization of these differentially expressed genes will be useful in elucidating the genetic basis of aging‐related changes in the submandibular salivary gland.

## 1 INTRODUCTION

Salivary glands are involved in the secretion of saliva, which participates in the protection and hydration of mucosal structures within the oral cavity, oropharynx, and esophagus, the maintenance of tooth integrity, antimicrobial defense, and protection from chemical and mechanical stress (Atkinson & Wu, 1994; Mandel, 1989). Saliva contains biologically active peptide and hormones, including digestive enzymes such as amylase, anti‐microbial substances such as secretory immunoglobulins, histatins, and growth factors such as epidermal growth factor (EGF) and nerve growth factor (NGF) (Nori et al., 2008). Aging affects the morphology and function of salivary glands, resulting in manifestations of dry mouth in elderly people (Barka, 1980). Many previous studies have described age‐related differences in the rate of flow and volume of saliva, as well as the contents of saliva (Melvin, Yule, Shuttleworth, & Begenisich, 2005; Nagler, 2004). In addition, age‐related changes in the salivary glands are consistently associated with a reduction of total and/or secretory protein synthesis (Nakamoto et al., 2007). However, the extent to which genetic alterations affect the function of these glands is unclear.

The klotho gene plays a critical role in regulating aging and the development of age‐related diseases in mammals. Life span is extended by up to 30% in transgenic mice over‐expressing the klotho gene compared with wild‐type mice (Kuro‐o, 2009; Kuro‐o et al., 1997). Klotho‐deficient phenotypes include osteoporosis, skin atrophy, ectopic calcification, pulmonary emphysema, hypogonadism, impaired bone mineralization, and neurodegeneration, which are also observed in the human aging phenotype (Kuro‐o et al., 1997). Many researchers have recently demonstrated an association between single nucleotide polymorphisms of the klotho gene and age‐related disorders, including coronary artery disease, senile osteoporosis, and stroke (Kawano et al., 2002; Ogata et al., 2002).

A few reports have shown a lack of eosinophilic granules and diminished granular ducts and lobes in the submandibular salivary glands of klotho‐deficient mice (Suzuki, Amizuka, Noda, Amano, & Maeda, 2006). In addition, histological observations have shown that the numbers of NGF‐ and EGF‐immunopositive ducts in the submandibular salivary gland are decreased in klotho‐deficient mice compared to wild‐type mice (Suzuki et al., 2006). However, these gene depletion studies have not provided insights on the regulation of salivary gland function during aging.

The purpose of the present study was to evaluate and compare differences in gene expression associated with aging in the submandibular salivary gland of klotho‐deficient mice. We used DNA microarray platforms in combination with functional signaling pathway analysis in this study. In addition, the data provided a network for investigating PARP‐γ transcriptional programs in the submandibular salivary gland in klotho‐related aging. These differentially expressed genes will contribute to an understanding of the genetic basis of klotho and the elucidation of the mechanism of biological behavior in the submandibular salivary gland.

## 2 MATERIALS AND METHODS

### 2.1 Animal models and genotyping assay for klotho gene

Experiments were performed in accordance with the Animal Research Institute Committee of Chosun University for the Care and Use of Laboratory Animals. All mice were generated by mating pairs of heterozygous klotho mice (Kl±) generously provided by Dr. Kuro‐o (University of Texas Southwestern, Dallas, TX, USA), and their genotypes were verified by PCR using genomic DNA extracted from the tail. For genotyping PCR analysis, 1–2 mm sections of tail were dissolved in 0.1 ml of 50 mM Tris (pH 8.0), 100 mM EDTA, 0.5% SDS, and 0.5 mg/ml proteinase K (Roche) solution at 55°C for at least 1 hr with vigorous shaking. The DNA was purified by phenol/chloroform extraction followed by ethanol precipitation and then dissolved in 0.1 ml of TE solution. We used the following specific primers: wild‐type klotho, forward 5′‐TTGTGGAGATTGGAAGTGGACGAAAGAG‐3′ and reverse 5′‐CTGGACCCCCTG‐AAGCTGGA‐GTTAC‐3′; klotho mutant, forward 5′‐TTGTGGAGATTGGAAGTGGACGAAAGAG‐3′ and reverse 5′‐CGCCCCGACCGGAGCTGAGA‐GTA‐3′. The GAPDH PCR primers were forward 5′‐CCAAGGTCATCCATGACAACT‐3′ and reverse 5′‐GCATTGCTGATGATCTTGAGGCTG‐3′. These primers were expected to produce 815 bp (WT) and 419 bp (klotho‐deficient) amplification products. The PCR conditions were as follows: denaturation at 94°C for 5 min, 30 cycles of 94°C for 30 s, annealing at 60°C for 1 min, and extension at 72°C for 45 s, and a final extension at 72°C for 10 min.

### 2.2 Cell culture

Human submandibular gland cells (HSG) were maintained in complete medium comprising Dulbecco's modified Eagle's medium (DMEM), 10% fetal bovine serum, 100 units/ml penicillin and 100 μg/ml streptomycin. Immortalized human salivary gland acinar cells (AC) were cultured on keratinocyte serum‐free medium (K‐SFM, Gibco/Life Technologies, Grand Island, NY) containing 100 unit/ml penicillin and 100 μg/ml streptomycin. The cells were maintained at 37°C in an humidified 5% CO_2_/95% air atmosphere.

### 2.3 Tissue preparation and histological examination

At 4 weeks of age, all animals were killed under ether anesthesia, and the submandibular salivary glands were dissected. The submandibular salivary gland tissue and tongue were fixed in 10% formalin, embedded in paraffin and cut into 4 μm‐thick sections for staining. All sections were stained with hematoxylin and eosin. Sections of tongue were also stained with von Kossa, Elastin, and Congo Red to detect histological alterations such as calcification, fibrosis, and amyloid accumulation.

### 2.4 RNA purification and RT‐PCR

Total RNA was isolated from the salivary glands of wild or klotho−/− mice (4 weeks old) using TRIzol reagent (Invitrogen, Calsbad, CA). To avoid genomic DNA contamination, the extracted RNA was purified using an RNeasy kit (Invitrogen). The quantity and quality of the RNA were determined by measuring the optical density (OD) at 260 and 280 nm. A 2 µg of RNA were used for cDNA synthesis using an oligo‐(dt)_15_ primer and M‐MLV reverse transcriptase. The reverse transcription (RT) reaction included an initial 10 min incubation at room temperature, followed by 60 min at 42°C and 10 min at 70°C to terminate the reaction. Subsequently, a 2 µl aliquot of cDNA was PCR amplified in a total volume of 25 µl containing 2.5 µl of 10 × PCR buffer (0.2 M Tris‐HCl (pH 8.4), and 0.5 M KCl), 0.2 mM dNTP mix, 1.5 mM MgCl_2_, 0.2 µM each primer, and 1.25 units of Platinum Taq DNA polymerase (Invitrogen). The thermal cycler profile was 95°C for 5 min, followed by 30 cycles of 95°C for 30 s, 55–60°C for 30 s, and 72°C for 30 s, with a final extension step at 72°C for 10 min. The specific primers for RT‐PCR are described in Table 1. The PCR products were then electrophoresed on a 2% acrylamide gel and visualized using a gel documentation system (Bio‐Rad, Hercules, CA).

**Table 1:** Primer sequences for RT‐PCR validation of the microarray data

Gene	Primer sequences (5′‐3′)	Gene	Primer sequences (5′‐3′)
Aifm 2	F: CCTGGCAAGTTTAACGAGGTGTC	FGFR1	F: GCGACTTCCATAGCCAGATGGCTG
	R: CCTGGCAAGTTTAACGAGGTGTC		R: TCGCCAAGTGGTTTGCCTAAGACC
Atp2a1	F: GAGCAGTTCGAAGACCTGCTTGTG	FGF15	F: ATACGGGCTGATTCGCTACTCGGA
	R: CCTGTCAGGATGGACTGGTCGA		R: TGAACGGATCCATGCTGTCAC
Atp1a2	F: TGCCATGGATGACCACAAGCTGTC	FGF23	F; GTCTGCAGCTTGGGCACTGCTA
	R: ACTACAGCCGCTAGCACGATACC		R: CACCAGGTAATGCTTCTGCGA
Aquaporin 3	F: AAGCTGCCCATCTATGCACTGGCA	Gpx3	F: AGTATGGAGCCCTCACCATCGA
	R: CAGTCGTGAAGACTTCTGAGC		R: CGCCTCATGTAAGACAGGATGTCC
Aquaporin 4	F: GCAGTGCTTTGGCCACATCAGTGG	Muc19	F: TGCTGGTTCCACATCTGCAAGAGC
	R: GTTCGTTGGAATCACAGCTGGCA		R: TTCGGTACAGGTACACTGATGGCA
Aquaporin 5	F: CATTGCTGGAGCAGGCATCCTGTA	Klk1b26	F: GTCGACCAGTGTGAGGTTTGGCTG
	R: CACGATCGGTCCTACCCAGAAGAC		R: GCCATCTTGTGGGTGTAATGCTGC
Cidea	F: CCTGCAGGAACTTATCAGCAAGAC	Klotho (Kl)	F: TGACCCGAATGTCTATCTGTGGGA
	R: TCGTGGCTTTGACATTGAGACAGC		R: GCACGATAGGTCATGTTCCGTGTG
CTGF	F: TGAGTCCTTCCAAAGCAGCTGCAA	Lcn2	F: TGGCAGGCAATGGGCTCCAGAA
	R: AACTCGGGTGGAGATGCCCATTCC		R: TGGCGAACTGGTTGTAGTCCGTG
Cxcl9	F: GGGCATCATCTTCCTGGAGCAGTG	Muc1	F: GCAGTTCCTTAGCATCGACTACCA
	R: ACATTTGCCGAGTCCGGATCTAGG		R: GAGGTGCTACTATGGTCTGGAG
Cox8b	F: TGCGAAGTTCACAGTGGTTCCCAA	NGFb	F: AGCATGGTGGAGTTTTGGCCTGTG
	R: CAAGTGGGCTAAGACCCATCCTGC		R: GTCCACAGTGATGTTGCGGGTCTG
EGF	F: ACGGTCAGGATTAACCTCCATCCA	Trpv1	F: GCCTGAAGCAGTTTGTCAATGCCA
	R: GCTGCATCCACCATTGTCAGGCGA		R: ACGAACTTGGTGTTGTCAGCTGTG
EGFBP2	F: GTCGACCAGTATGAGGTTTGGCTG	Ucp1)	F: GACACTGCCAAAGTCCGCCTTCAG
	R: TGGCACAGTTCTCATTGGGCA		R: TGTAGGCTGCCCAATGAACACTGC
FABP3	F: AGTCACTCGGTGTGGGCTTTGCCA	VCAM1	F: CCAAGTCCGTTCTGACCATGGAGC
	R: GAGTGCTCACCACACTGCCATGAG		R: TCATGAGCTGGTCACCCTTGAA

### 2.5 Microarray raw data preparation and statistical analysis

Total RNA was amplified and purified using an Ambion Illumina RNA amplification kit (Ambion, Austin, TX) to obtain biotinylated cRNA according to the manufacturer's instructions. The array signal was detected using an Illumina MouseRef‐8 v2 Expression BeadChip (Illumina, Inc., San Diego, CA) following the bead array manual. The arrays were scanned with an Illumina bead array reader confocal scanner according to the manufacturer's instructions. Array data export processing and analysis were performed using Illumina BeadStudio v3.1.3 (Gene Expression Module v3.3.8, Illumina, Inc.). The quality of hybridization and overall chip performance were monitored by visual inspection of both internal quality control checks and raw scanned data. The raw data were extracted using the software provided by the manufacturer (Gene Expression Module v1.5.4, Illumina, Inc., San Diego, CA). The array data were filtered by a detection *p*‐value <0.05 (similar to signal to noise) in at least 50% samples (we applied a filtering criterion for data analysis; a higher signal value was required to obtain a detection *p*‐value <0.05). The selected gene signal value was transformed by logarithm and normalized by the quantile method. Comparative analysis between the control group and test group was performed using fold‐change. Biological ontology‐based analysis was performed using the Panther database (http://www.pantherdb.org).

### 2.6 Functional and network analysis

We used Ingenuity Pathway Analysis (Ingenuity Systems, Inc., Redwood city, CA) to determine the statistically significant pathways, functions, and networks in which the identified genes regulated by klotho may be involved. Fisher's exact test was used to identify the significant functions and pathways represented within the respective gene sets.

## 3 RESULTS

### 3.1 Histological differences in the salivary glands of klotho‐deficient mice

**Figure 1:**
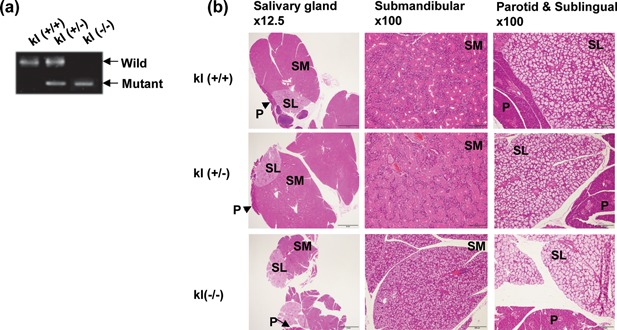
Histological features of the salivary gland in klotho−/− mice. (a) PCR genotyping of klotho‐deficient mice. Genomic DNA from mouse tails was used to amplify the fragments derived from the wild‐type and mutant alleles using two specific primers. (b) Staining with hematoxylin and eosin (×12.5 and ×100 magnification). Photomicrographs of the salivary glands in klotho wild‐type (kl+/+), hetero (kl±), and klotho‐deficient mice (kl−/−). SM, submandibular gland; SL, sublingual gland; P, parotid gland

First, to confirm the depletion of klotho gene expression in the salivary glands of Klotho mutant mice, we performed RT‐PCR analysis. As shown in Figure 1a, the mRNA expression levels of Klotho were suppressed in the salivary glands of Klotho mutant mice compared with wild‐type mice.

Histological analysis of the salivary glands of wild‐type and klotho‐deficient mice was performed at 4 weeks of age. In the klotho wild‐type and hetero mice, normally duct cells were found in the submandibular salivary gland, and in particular, there were many granular convoluted tubules with abundant granules. Compared to wild‐type mice, klotho‐deficient mice had less tubules and connective tissue in the submandibular salivary gland. In addition, the submandibular salivary gland was composed of serous acini and mucous acini in the wild‐type mice. However, in the klotho‐deficient mice, the submandibular salivary gland was composed of only mucous acini, and no serous acini were present (Figure 1b).

### 3.2 Strategy to identify genes related to salivary gland dysfunction in klotho−/− aging mice

The aim of this study was to identify salivary gland dysfunction‐related gene alterations in accelerated aging klotho−/) mice. The sample sets were as follows: klotho+/+ (WT), klotho± (HT), klotho−/− (MT). The gene expression data from all samples were obtained, quality control steps were performed, and the data were analyzed using Illumina BeadStudio v3.1.3. Genes related to submandibular salivary gland dysfunction in klotho−/− mice were identified by performing gene expression analysis followed by Gene Ontology (GO) analysis.

**Figure 2:**
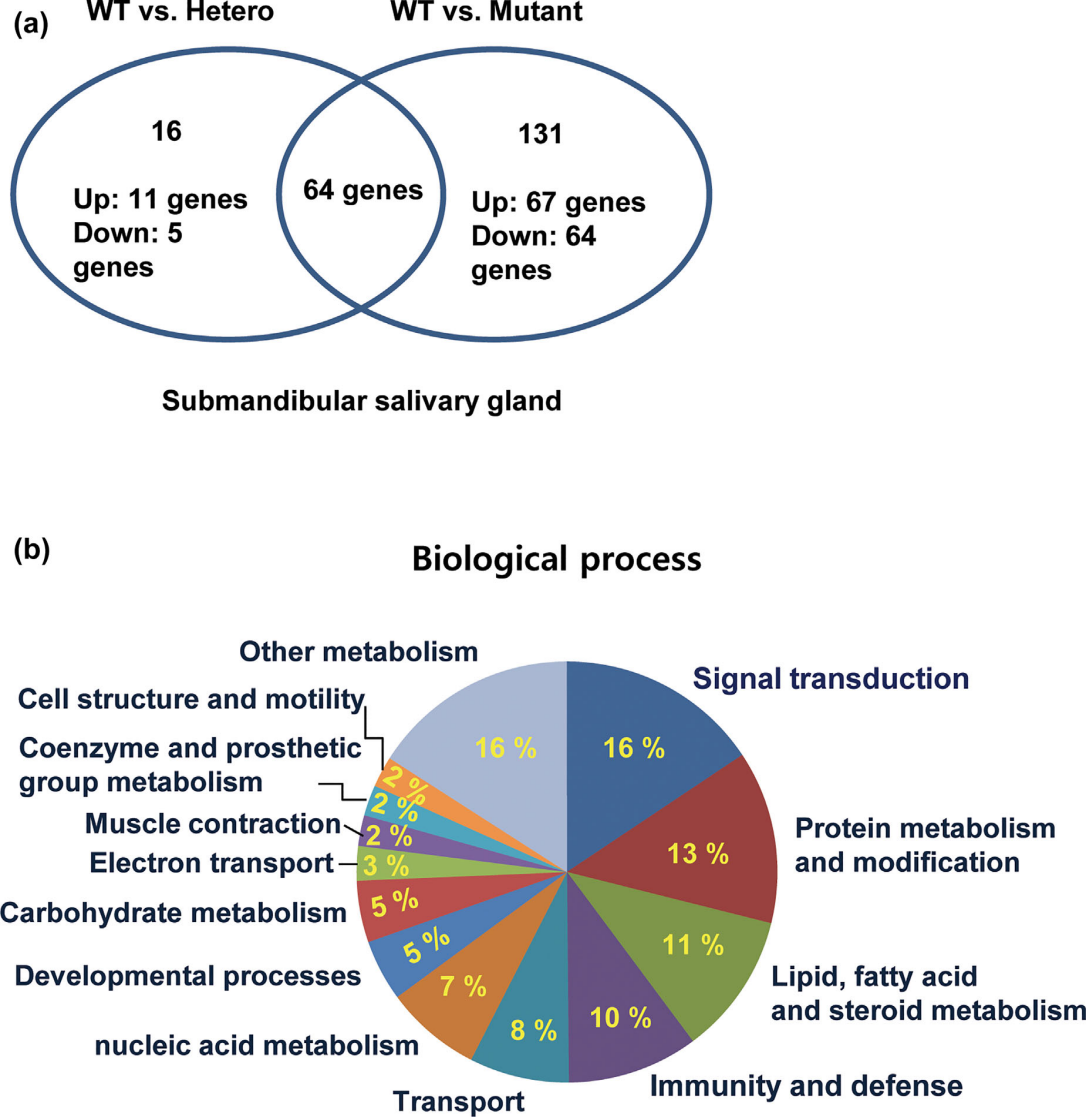
Gene ontology (GO) analysis of the altered genes in the submandibular gland of klotho‐deficient mice. (a) Venn diagrams showing the number of genes identified as genuinely regulated in klotho wild‐type versus klotho‐deficient mice. (b) The differentially expressed transcripts mapped to numerous biological processes of the hierarchical GO system. The gene expression ratio (≥2‐fold) was evaluated from gene expression profiles in the submandibular glands of wild‐type and klotho‐deficient mice

Differential gene expression analysis was performed by comparing the klotho+/+ and klotho± mice or klotho+/+ and klotho−/− mice. All genes with *p*‐value <0.05 and fold‐change in expression ≥2.0 were considered statistically significant. The majority of genes displayed changes in expression levels in the submandibular salivary gland in klotho −/− compared with klotho wild‐type. The expression levels of 64 genes decreased by more than twofold in klotho −/−, whereas 67 genes exhibited increases in expression of greater than twofold in klotho−/− mice, based on statistical group comparison between the wild‐type and klotho‐deficient mice. The statistical analysis of the data generated a set of 131 genes, and the top 20 up‐ or down‐regulated genes are shown in Tables 2 and 3. In the Venn diagram, among a total of 195 genes (klotho+/+ vs. klotho−/−), 64 genes were present in klotho+/+ versus klotho± mice (Figure 2a).

**Table 2:** Top 20 genes differentially up‐regulated in the salivary gland of klotho wild‐type mice versus klotho‐deficient mice

Refseq	Gene symbol	Fold change	Regulation	Gene description
NM_011414	SLPI	34.34	Up	Antileukoproteinase
NM_027222	2010001M09Rik	9.64	Up	RIKEN cDNA 2010001M09 gene
NM_011066	PER2	8.21	Up	Period circadian protein homolog 2
NM_001012	MUP20	6.22	Up	Major urinary protein 20
NM_008647	MUP2	5.68	Up	Major urinary protein 2
NM_026929	CHAC1	5.67	Up	Cation transport regulator‐like 1
NM_198091	USP2	5.46	Up	Ubiquitin carboxyl‐terminal hydrolase 2
NM_029720	CRELD2	4.38	Up	Cycteine‐rich with EGF‐like domain protein 2
NM_013650	S100A8	4.03	Up	S100 calcium‐binding protein A8
NM_008039	FPR2	3.93	Up	Formyl peptide receptor 2
NM_011136	POU2AF1	3.64	Up	POU domain class 2‐associating factor 1
NM_010220	FKBP5	3.64	Up	FK506‐binding protein 5
NM_001039	COQ10B	3.57	Up	Coenzyme Q10 homolog B
NM_010217	CTGF	3.50	Up	Connective tissue growth factor
NM_025290	RSPH1	3.18	Up	Radial spoke head 1 homolog
NM_008491	LCN2	3.13	Up	Lipocalin 2
NM_009787	PDIA4	3.12	Up	Protein disulfide isomerase family A
NM_031188	MUP1	2.93	Up	Major urinary protein 1
NM_009251	SERPINA3G	2.82	Up	Serine protease inhibitor A3G
NM_007812	CYP2A5	2.68	Up	Cytochrome P450, family 2, subfamily a, polypeptide 5

**Table 3:** Top 20 genes differentially down‐regulated in the salivary gland of klotho wild‐type mice versus klotho‐deficient mice

Refseq	Gene symbol	Fold change	Regulation	Gene description
NM_023186	CHIA	−20.30	Down	Chitinase, chitin, and chitotriose degradation
NM_010644	KLK1B26	−17.33	Down	Kallikrein 1‐related peptidase b26
NM_007751	COX8B	−15.35	Down	Cytochrome c oxidase subunit VIIIB
NM_009463	UCP1	−14.85	Down	Uncoupling protein 1, proton carrier
NM_010642	KLK1B21	−14.29	Down	Kallikrein 1‐related peptidase b21
NM_010915	KLK1B4	−14.00	Down	Kallikrein 1‐related peptidase b4
NM_013645	PVALB	−13.44	Down	Parvalbumin
NM_021285	MYL1	−12.04	Down	Myosin light chain 3 skeletal muscle isoform
NM_010116	KLK1B9	−11.48	Down	Kallikrein 1‐related peptidase b9
NM_009606	ACTA1	−11.17	Down	Actin, alpha skeletal muscle
NM_010115	EGFBP2	−10.92	Down	Epidermal growth factor binding protein type b
NM_007702.1	CIDEA	−10.21	Down	Cell death‐inducing DFFA‐like effector a
NM_198669	PRB1	−9.73	Down	Basic salivary proline‐rich protein 1
NM_010174	FABP3	−9.47	Down	Fatty acid binding protein 3
NM_001004	Gm5154	−8.96	Down	Predicted gene 5154
NM_011174	PRH1	−8.73	Down	Proline‐rich protein haelll subfamily 1
NM_010645	KLK1B1	−8.16	Down	Kallikrein 1‐related peptidase b1
NM_009394	TNNC2	−7.86	Down	Troponin C type2
NM_031499	PRP2	−7.17	Down	Mus musculus proline‐rich protein 2
NM_001024	PRPMP5	−6.44	Down	Proline‐rich protein MP5

To further investigate the biological function classifications of the genes related to submandibular salivary gland dysfunction in klotho‐deficient mice, we performed GO analysis of all sets of genes regulated in klotho WT versus klotho−/− mice. Among the identified genes, signal transduction (16%), protein metabolism and modification (13%), lipid, fatty acid and steroid metabolism (11%), immunity and defense (10%), and transport (8%) were over‐represented (Figure 2b). Molecular function analysis revealed that, in klotho−/− mice, many pathway genes were differentially expressed, especially in the transferase (9%), oxidoreductase (9%), protease (9%), receptor (7%), and transcription factor (6%) categories (data not shown).

### 3.3 Validation of the microarray results

**Figure 3:**
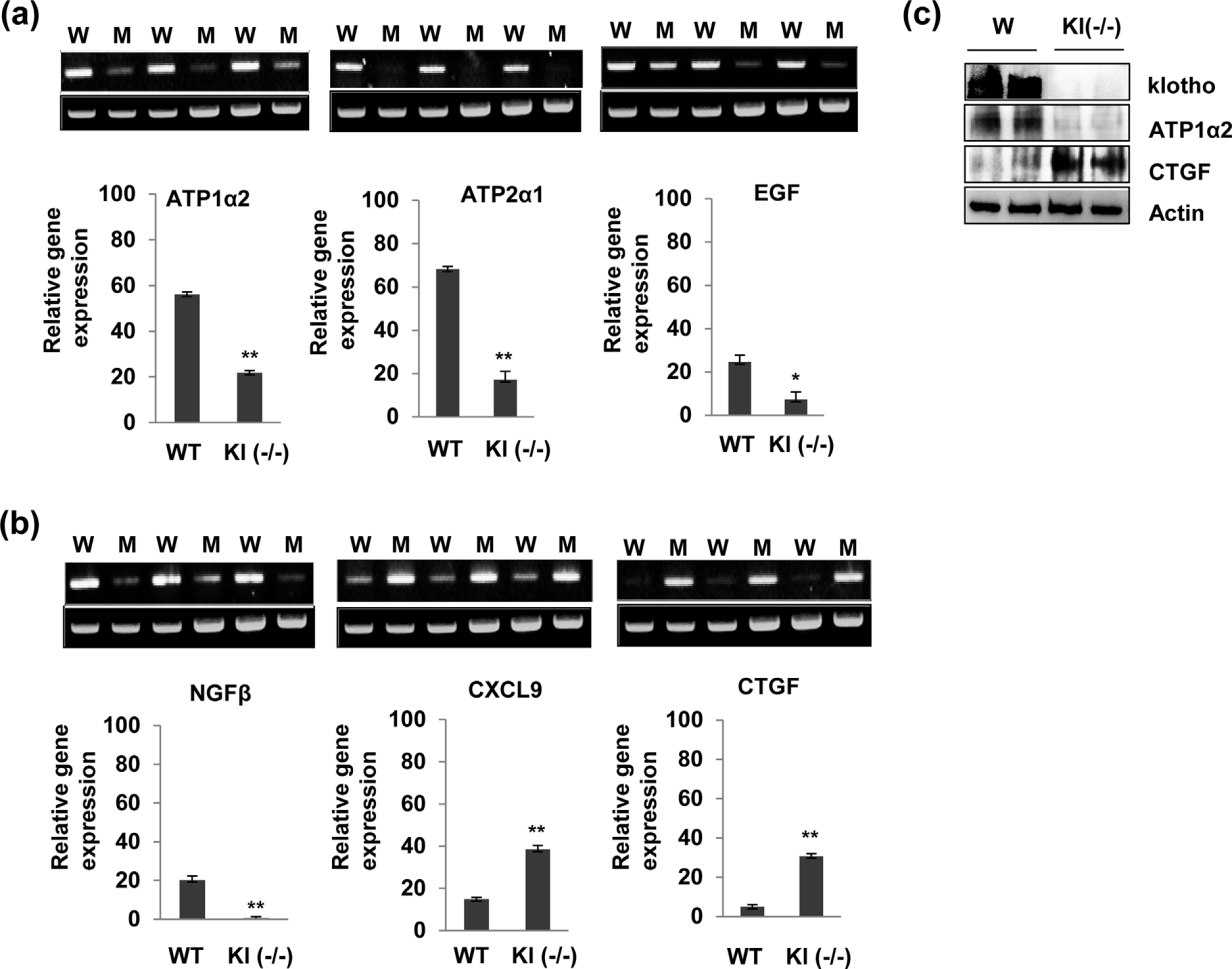
Comparison of gene expression in wild‐type and klotho‐deficient mice. (a,b) Total RNAs were extracted from submandibular gland tissues isolated from individual mice. cDNA was synthesized by reverse transcription‐polymerase chain reaction (RT‐PCR). mRNA levels were normalized to GAPDH. The bar graph represents expression relative to GAPDH. The data are reported as the mean ± SD of three independent experiments. *p < 0.05, **p < 0.001. (c) Expression of klotho, ATP1α2, and CTGF protein in submandibular gland tissues of wild‐type and klotho‐deficient mice. The total protein was extracted, and klotho, ATP1α2, and CTGF protein levels were measured by Western blot, respectively. Actin was used as a loading control

Quantitative reverse transcription PCR (qRT‐PCR) was used to validate the alterations in gene expression detected in klotho−/− mouse submandibular tissues using microarray analysis. The mRNA expression levels of six selected genes in submandibular tissues from wild‐type and klotho‐deficient mice were showed by RT‐PCR analysis. The mRNA expression levels of the Egf, Ngf, Atp1a2, Atp2a1, and Gpx3 genes significantly decreased in klotho‐deficient mice compared with wild‐type mice. However, the Cxcl9 and Ctgf genes significantly increased in klotho‐deficient mice (Figures 3a and 3b). Immunoblotting constantly showed that klotho and ATP1α2 were upregulated in submandibular tissues of wild‐type mice. The level of CTGF protein was downregulated in wild‐type mice compared with klotho‐deficient mice (Figure 3c).

### 3.4 Ingenuity pathway analysis in klotho−/− mice submandibular salivary dysfunction

**Figure 4:**
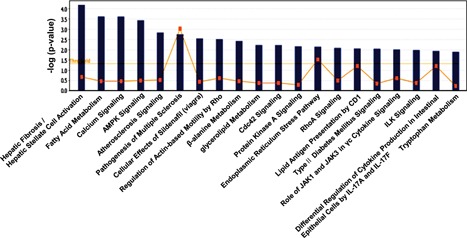
Ingenuity Pathway Analysis of the genes that were regulated in the klotho‐deficient salivary gland. (a) The significance of each function or canonical pathway was determined based on the *p*‐values determined using Fisher's exact test and a threshold less than 0.05. The top 20 possible functions and canonical pathways are shown

**Figure 5:**
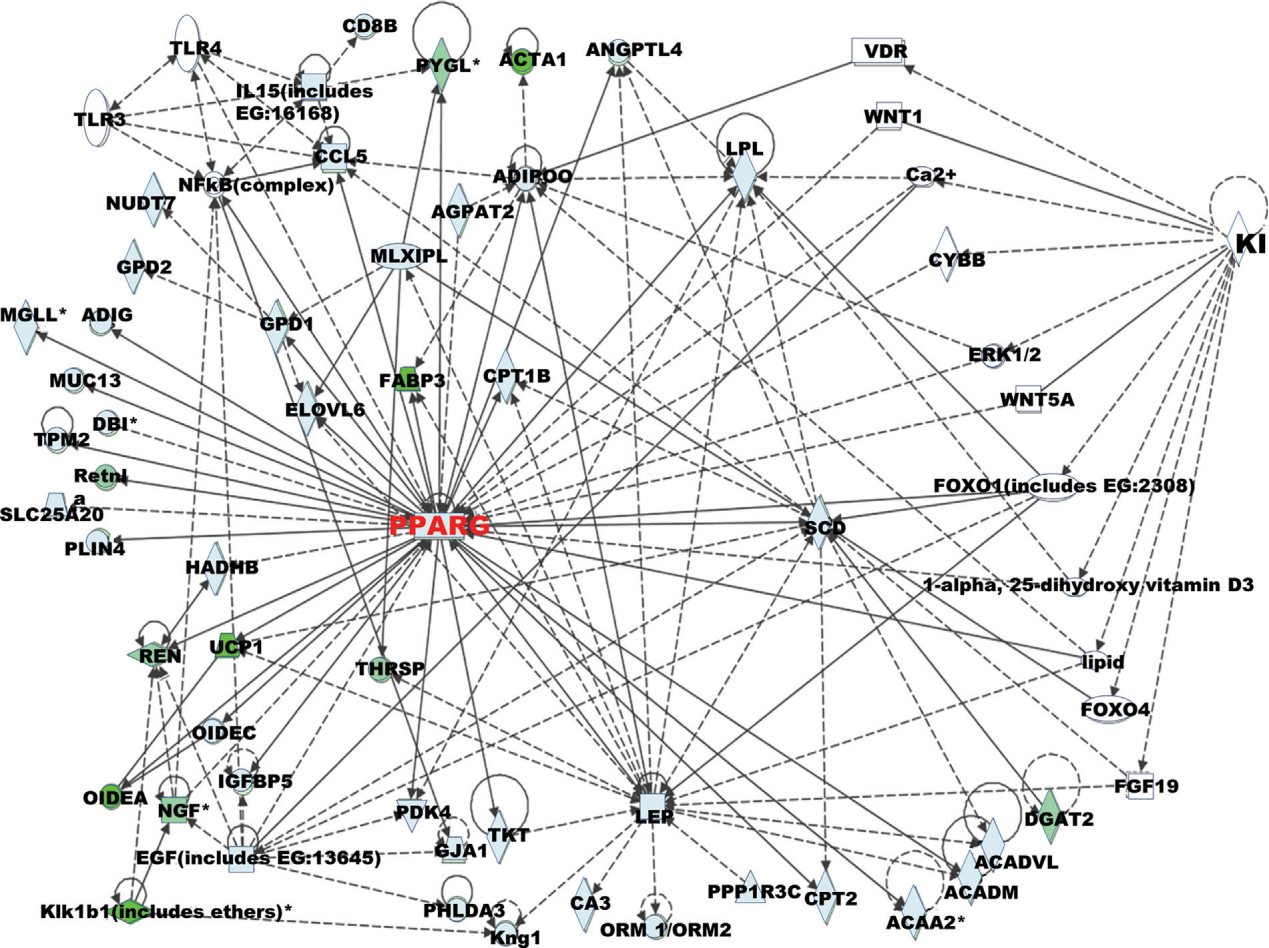
Network connectivity of differentially regulated genes in the salivary glands of klotho‐deficient mice. Ingenuity Pathway Analysis was applied to genes showing significant dysregulation, with filtering on their relative distance from the mean ratio of the population. The first main pathway that appeared to be differentially expressed was PPAR γ signaling. Genes belonging to this pathway were significantly differentially down‐regulated

Ingenuity pathway analysis (IPA) was performed on all genes identified as regulated in the submandibular salivary gland in klotho−/− mice. Fisher's exact test was applied, and we identified the top 20 significant canonical pathways based on *p*‐value <0.05 and a threshold value of log (*p*‐value) of 0.05. The significant pathways, which included fatty acid metabolism, calcium signaling, AMPK signaling, endoplasmic reticulum stress pathway, glycerolipid metabolism, and type II diabetes mellitus signaling, are shown in Figure 4. The most significant canonical pathway was energy metabolic signaling, followed by lipid metabolism, glycerolipid metabolism, hepatic fibrosis, and differential regulation of cytokine production in intestinal epithelial cells. Among the genes belonging to energy metabolic signaling, peroxisome proliferator‐activated receptor gamma (PPAR γ), which is a ligand‐activated transcription factor that mainly regulates genes responsible for cellular differentiation, development, fatty acid (FA) storage, and energy metabolism, was significantly differentially expressed between klotho+/+ and klotho−/− mice. The expression of this gene was 2.4‐fold lower in klotho−/− mice compared with klotho+/+ mice (Table 4). We specifically focused on several genes linked to the PPAR γ networks. Gene network analysis also revealed overlapping network connectivity for 42 of the differentially expressed genes in the klotho−/− submandibular gland (Figure 5).

**Table 4:** Identification of differentially expressed transcription factors in the salivary gland of klotho‐deficient mice

Transcription regulator	*p*‐value of overlap	Regulation z‐score	Predicted activation state
PPARG	1.61E‐09	−2.416	Inhibited
PPARA	1.70E‐08	−2.967	Inhibited
NFE2L2	4.79E‐06	0.715	
CEBPA	1.51E‐05	−0.381	
MYOD1	7.61E‐05	−2.020	Inhibited

### 3.5 **Gene expression of the PPAR** γ **pathway in the klotho−/− salivary gland**

**Figure 6:**
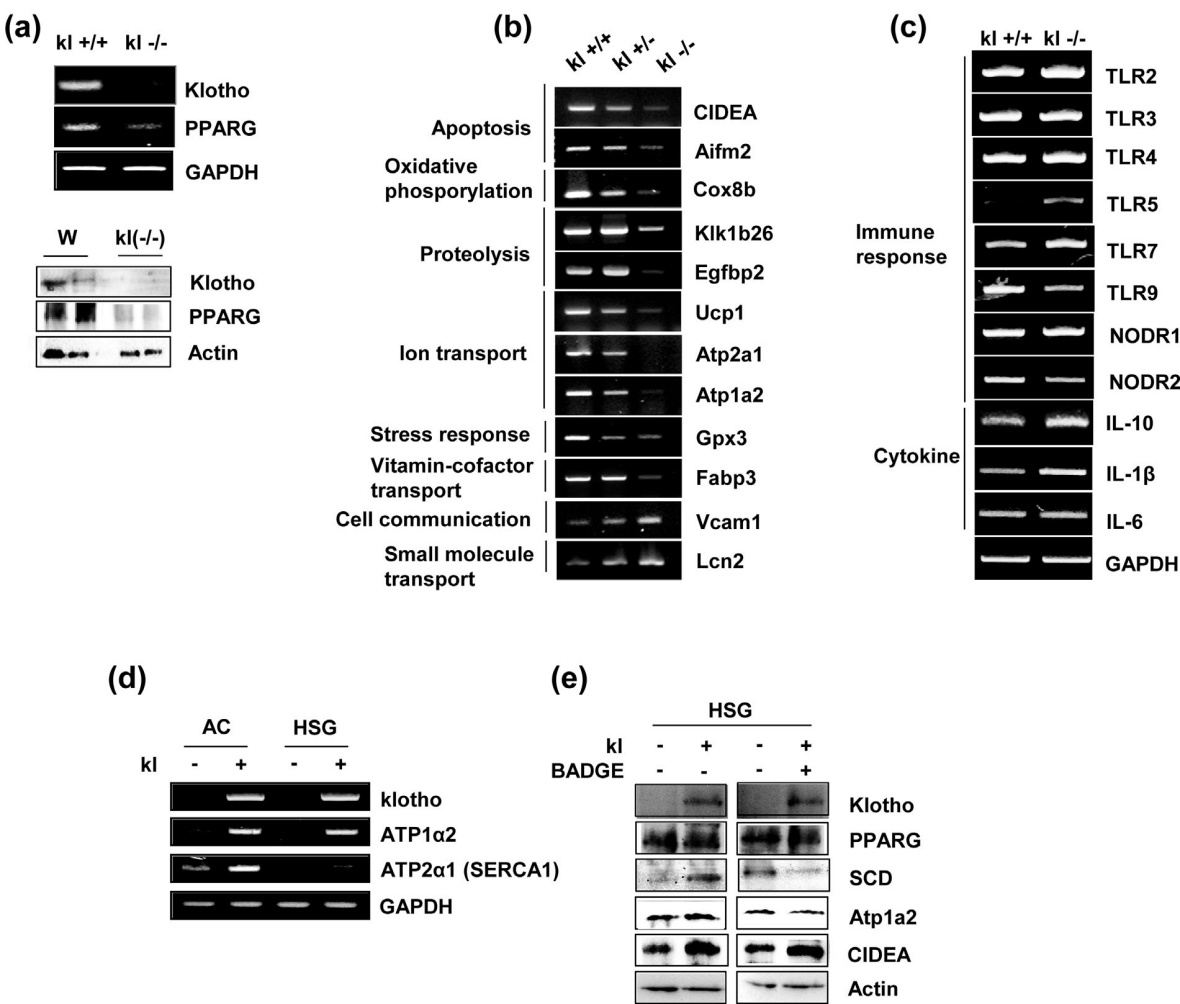
Validation of genes expression belonging to the PPAR γ pathway in the salivary glands of mice. mRNA and protein were extracted from the salivary glands of klotho wild‐type (Kl+/+), hetero (Kl±), and klotho‐deficient mice (Kl−/−). *(*a) RT–PCR and Western blot analysis of klotho and PPARG in klotho wild‐type (Kl+/+) and klotho‐deficient mice (Kl−/−). (b) The mRNA expression of genes related to the PPAR γ pathway. (c) Validation of toll‐like receptor (TLR) genes. RT–PCR was performed using the primers described in Table 1. (d) Expression of endogenous ATP1α2 and ATP2α1 (SERCA1) in klotho‐overexpressing AC and HSG salivary gland cells. Cells were transfected with klotho expression plasmids. A total of 48 hr after transfection, total RNA was prepared and subjected to RT‐PCR. (e) HSG cells were transfected with pcDNA3.1‐klotho for 24 hr, treated to PPARG antagonist BADGE (30 µM) and incubated for another 20 hr. A Western blot analysis was performed to assess the PPARG, SCD, ATP1α2, and CIDEA levels

PPARs mainly control the expression of gene networks involved in adipogenesis, lipid metabolism, anti‐inflammation, and the maintenance of metabolic homeostasis (Echeverría, Ortiz, Valenzuela, & Videla, 2016). To further substantiate the differences in the DNA microarrays and the expression levels of PPAR γ and genes related to the PPAR γ pathway, we performed RT‐PCR and Western blot analysis. The mRNA and protein of PPAR γ were down‐regulated in klotho‐deficient submandibular tissues (Figure 6a). PPAR γ‐related genes involved in “signaling transduction,” especially oxidative phosphorylation (Cox8b), proteolysis (Klk1b26 and Egfbp2), ion transport (Atp1α2 and Atp2α1), and stress response (Gxp3), were down‐regulated. By contrast, the expression of genes (Lcn2, IL‐10, and IL‐1β) involved in “small molecular transport and cytokine” was increased in klotho−/− mice (Figure 6b). In addition, several key players in the water channel (Aqp3, Aqp4, and Aqp5) and endocrine signaling (FGF15 and FGF23) were differently regulated in klotho‐deficient submandibular tissues (data not shown). The changes in the expression of TLR5, TLR7, TLR9, and NODR2, toll‐like receptor (TLR) genes that play a key role in the innate immune system, were also validated (Figure 6c). No significant changes were seen in the expression of TLR2, TLR3, TLR4, and TLR7.

To investigate the molecular mechanism underlying the PPARG function in klotho overexpressed AC and HSG cells, the mRNA expression levels of ATP1α2 and ATP2α1, a known transcriptional target of PPARG, was analyzed. We observed higher levels of klotho in the klotho‐transfected cells compared with the control cells. Remarkably, the overexpression of klotho caused increased expression of ATP1α2 and ATP2α1 mRNA in AC and HSG cells (Figure 6d).

We next evaluated the effect of the PPARG antagonist BADGE in klotho‐overexpressed HSG cells. Results revealed that Klotho induced the expression of target proteins of PPARG such asSCD, ATP2α1, and CIDEA, compared to control cells. In this study, the expression of SCD and ATP1α2 partially inhibited by PPARG antagonist BADGE treatment (Figure 6e). However, BADGE did not affect CIDEA expression in klotho overexpressed HSG cells. These results indicated that use of a PPARG antagonist partly affected the mechanisms of klotho‐mediated fatty acid and/or water channel.

### 3.6 Histological differences in the tongues of klotho‐deficient mice

**Figure 7:**
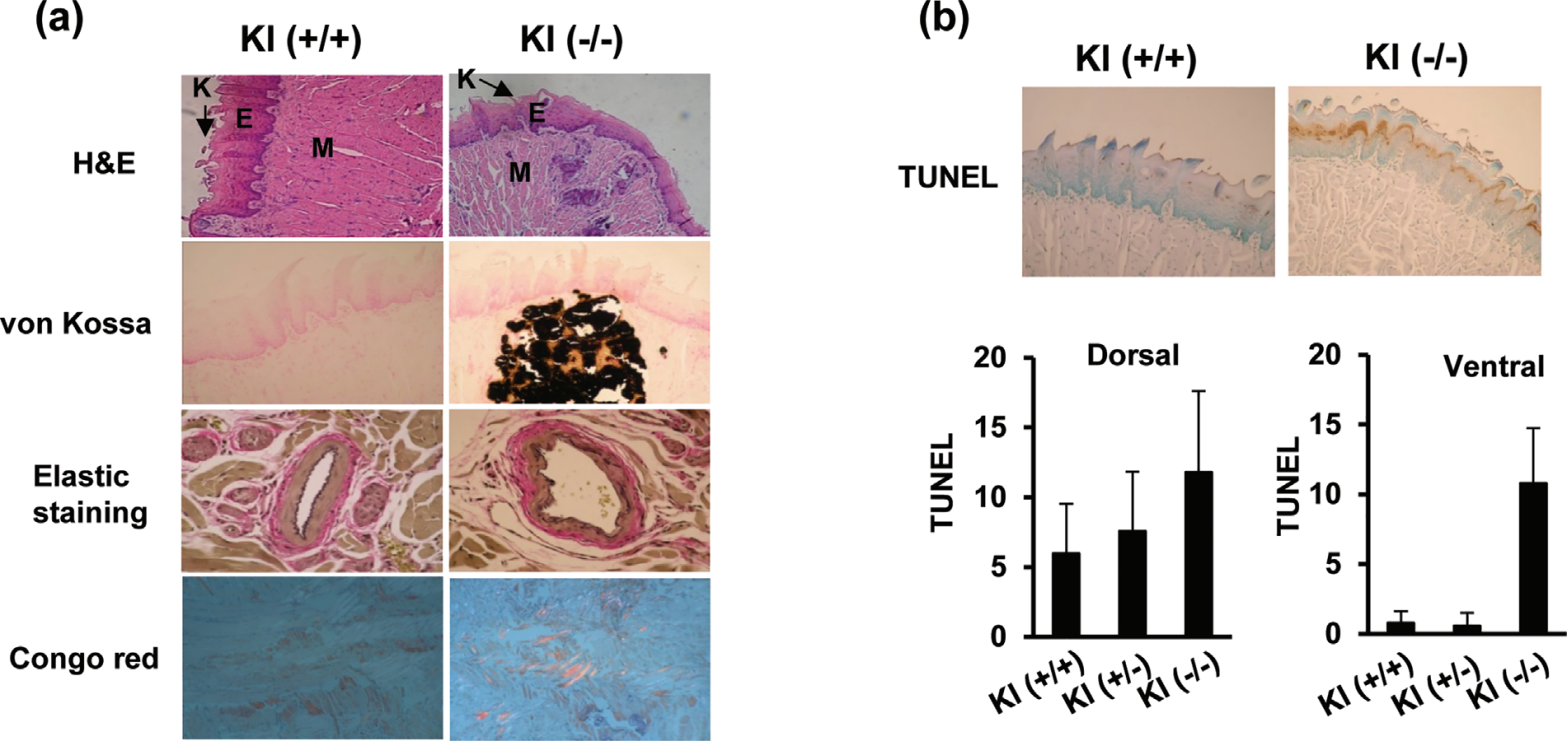
Histological features of the tongue in klotho−/− mice. (a) Staining with hematoxylin and eosin (×100 magnification). Photomicrographs of the tongues of klotho wild‐type (Kl+/+) and klotho‐deficient mice (Kl−/−). FP, filiform papillae; E, epithelium; M, muscle. Tongue tissue sections were evaluated using von Kossa, Congo red (CR), and elastin staining. (b) TUNEL assays were performed on paraffin sections from the tongues (dorsal or ventral) of klotho‐deficient mice. The TUNEL‐positive cells were counted, and the results are expressed. The data are reported as the mean ± SD of three independent experiments

To investigate the effect of salivary gland dysfunction on tongue morphology, we analyzed the tongues of klotho+/+ and klotho−/− mice (4 weeks). Histological sections of tongue were stained with hematoxylin‐eosin and subjected to von Kossa staining, elastin staining, Congo red staining, and TUNEL staining. As illustrated in Figure 7a, excessive calcification was observed in the tongue muscle of klotho−/− mice. Therefore, increased elastin fiber in the blood vessel wall and amyloidosis were observed in the tongues of klotho−/− mice compared to klotho+/+ mice. Tongue sections were then analyzed by TUNEL for apoptotic cells. The klotho*−/−* mice exhibited an increased number of TUNEL‐positive cells, and the quantitative analysis of TUNEL‐positive cells in the dorsal and ventral portions of the tongue is summarized in Figure 7b.

## 4 DISCUSSION

We have performed gene profiling of the salivary gland to provide a database for the interpretation of age‐dependent alterations. Because older healthy individuals who do not use medications exhibit salivary gland dysfunction, such as a lower resting salivary flow rate, compared with younger individuals, there is an urgent need to detect and identify proteins that regulate salivary gland function under aging conditions. In this investigation, we analyzed gene expression in mouse submandibular glands using cDNA microarray and assessed the microarray results by semi‐quantitative RT‐PCR in accelerated aging klotho‐deficient mice. The semi‐quantitative RT‐PCR results for six genes were largely consistent with the results of the microarray analysis. Therefore, the results for the large number of genes in the cDNA microarray analysis may be useful for comparing gene expression in the salivary gland between wild‐type and klotho‐deficient mice.

The submandibular salivary glands have been considered an age‐stable organ (Ramirez & Soley, 2011). However, we and others have reported that submandibular salivary glands in accelerated aging klotho‐deficient mice show a loss of granular ducts and mucous acini compared to wild type mice (Suzuki et al., 2006). Except for a loss of ducts in the submandibular salivary gland, we observed a comparatively stable structure of the submandibular gland, parotid, and sublingual gland in aging klotho‐deficient mice. The mouse submandibular gland contains various biological molecules such as epidermal growth factor (EGF), nerve growth factor (NGF), renin, kallikreins, and proteases (Atkinson & Wu, 1994; Sabbadini & Berczi, 1995). EGF and NGF have been reported to be biosynthesized in granular convoluted tubule (GCT) cells of the submandibular ducts and secreted in the saliva of mice (Gresik, 1994). A previous study showed that the granular ducts of the salivary glands exhibited remarkably decreased immunoreactivities for NGF and EGF in klotho‐deficient mice (Suzuki et al., 2006). Interestingly, we also confirmed that the expression of EGF and NGF was inhibited in the submandibular glands of klotho‐deficient mice.

Our data analysis suggested that ATP1α2 and ATP2α1/SERCA1, subtypes of Na^+^/K^+^‐ATPase and Ca^2+^ATPase, were downregulated in the submandibular salivary glands of aging Klotho‐deficient mice. In the salivary glands, ATP1α2, a subtype of Na^+^/K^+^‐ATPase, is localized mainly at basal infolding of the striated duct and excretory ducts, though it is also weakly expressed on the membranes of granular convoluted tubules (GCT) and on acinar basolateral membranes (Sims‐Sampson, Gresik, & Barka, 1984). The activity of ATP1α2 establishes transmembrane ion gradients and is essential to cell function and survival (De Lores Arnaiz & Ordieres, 2014).

However, the link between disruption of Na^+^/K^+^‐ATPase activity and salivary gland dysfunction in aging remains to be clarified. The salivary fluid secretion is dependent on Cl^‐^ transport across the apical membrane of acinar cells. Intracellular accumulation of Cl^‐^ requires a Na^+^ gradient (Chaib, Kabre, Metioui, Franco, & Dehaye, 1999). Thus, saliva secretion from the salivary gland may be dependent on Na^+^/K^+^‐ATPase activity.

Additionally, in the salivary gland, saliva secretion is initiated by activation of phospholipase C, generation of inositol 1,4,5 trisphosphate (IP3), and release of Ca^2+^ from the ER to the cytosol. The cytosolic Ca^2+^ is taken up by sarco/endoplasmic reticulum Ca^2+^‐ATPases (SERCA1‐3) (Homann, Kinne‐Saffran, Arnold, Gaengler, & Kinne, 2006). The sarco/endoplasmic reticulum Ca^2+^‐ATPase (SERCA) is the active Ca^2+^ transporter in the sarcoplasmic reticulum (SR), and regulation of its function is a key mechanism of Ca^2+^ homeostasis and depends on the cell type and state of differentiation (Homann et al., 2006). A significant age‐dependent loss in Ca^2+^‐ATPase activity and Ca^2+^‐uptake rate has been observed specifically in the rat skeletal‐muscle SR (Schöneich, Viner, Ferrington, & Bigelow, 1999). Despite these changes in aging, whether Ca^2+^‐ATPase (SERCA) affects salivary gland function in aged mice is unclear. However, in the salivary gland, cytosolic calcium or sodium reduction may be important for salivary gland dysfunction during aging as well as in dystrophic pathological conditions. Further studies are needed to precisely elucidate the functional significance of changes in the ion efflux pump as well as salivary gland dysfunction both in aging and in diseases.

We also observed that CXCL9 was increased in the klotho−/− salivary gland. CXCL9 proteins are predominantly expressed in the ductal epithelium adjacent to lymphoid infiltrates in the Sjögren's syndrome salivary gland but are not expressed in the normal salivary gland (Ogawa, Ping, Zhenjun, Takada, & Sugai, 2002). Therefore, in our study, the up‐regulation of CXCL9 might reflect a higher proportion of T cells in aged inflammatory tissue compared with healthy controls. CXCL9 is a validated biomarker of the development of tissue dysfunction, suggesting an underlying pathophysiological relation between the levels of these chemokines and the development of aged salivary dysfunction.

In our downregulated gene lists (Table 3), we found that several Kallikrein‐related peptidases are strongly downregulated in klotho−/− salivary glands. Recent studies suggested that activated KLKs may degrade insulin‐like growth factor‐binding proteins and extracellular matrix proteins such as fibronectin, laminin, and type IV collagen (Dong et al., 2014; Hekim et al., 2010; Linardoutsos, Gazouli, Machairas, Bramis, & Zografos, 2014). Degradation of extracellular insulin‐like growth factor‐binding proteins would increase the concentration of free insulin‐like growth factor, and this could eventually stimulate cell growth and lifespan extension. In addition, IGF‐1 binding proteins are important is regulation of the IGF‐1 axis that also regulates peripheral glucose metabolism and body fat distribution.

We may suspect that KLKs is important in longevity mechanisms that link IGF signaling and aging with availability of energy resources. However, the current knowledge is insufficient to establish a precise, causal relationship between klotho and KLKs in aging phenotypes to which they contribute and to understand how biological specificity can be obtained. A comprehensive study of the expression patterns and/or function of klotho and KLKs in aging are needed to fill these gaps in the available knowledge.

Interestingly, IPA revealed that the deregulated genes in the klotho‐deficient mice submandibular gland are involved in a variety of pathways. Some of the major metabolic pathways are involved in fatty acid metabolism and calcium signaling, reflecting the cellular phenotype that accompanies activation of the peroxisome proliferator‐activated receptor. PPAR α and PPAR γ, member of the nuclear receptor family of transcription factors, play important roles in lipid and glucose metabolism, stress, and aging (Berger & Moller, 2002; Feige, Gelman, Michalik, Desvergne, & Wahli, 2006; Ulrich‐Lai & Ryan, 2013). PPAR γ agonists exhibit anti‐inflammatory activity by inhibiting cytokine production in a variety of mouse models for chronic inflammatory disease and immune disease, including multiple sclerosis (MS) and uveitis (Antonelli et al., 2014; Beauregard & Brandt, 2003; Shen et al., 2014). In addition, PPAR γ ameliorates Sjögren's syndrome through regulation of the expression of cytokines in peripheral blood and/or salivary gland in non‐obese diabetic mice (Li, Xu, Wang, & Wei, 2014). PPAR α and PPAR γ can inhibit IL‐1β‐induced NO production in cultured lacrimal gland acinar cells, suggesting that PPAR may be useful therapeutic target for preventing NO‐mediated gland damage. However, the effects of PPAR α and PPAR γ on the progression of aged salivary gland dysfunction are not clear.

Many specific genes targeting various metabolic pathways are modulated by both PPAR γ and PPAR α/γ in the aged klotho−/− salivary gland (Figure 5). Thus, many genes involved in PPAR‐targeted functions were regulated, including lipid metabolism (CIDEA, SCD, and Fabp3), chronic kidney disease (FGF23), ion transport (Atp2a1 and Atp1a2), mitochondrial oxidative phosphorylation (Cox8b), stress response (Gpx3), inflammation (IL‐10 and IL‐1β), immunity (TLR2‐9 and NODR1‐2), and water channel (AQP3‐5). Therefore, IPA and gene network analysis indicated that the nodal point in this cross‐talk in aged salivary gland dysfunction may be PPARα and/or PPARγ. Further studies are also needed to precisely elucidate the functional significance of changes in PARPs in aged‐salivary gland dysfunction.

The function of the salivary glands is to produce saliva, which is crucial for digestion, taste, the maintenance of tooth integrity, and anti‐microbial. Previous studies have indicated that anatomical changes in salivary gland with age is accompanied by atrophy of the acinar cells and replacement of the normal gland parenchyma with fibrous and/or adipose tissue (Azevedo, Damante, Lara, & Lauris, 2005; Choi, Park, Kim, Lim, & Kim, 2013; Syrjanen, 1984). A reduction in saliva leads to xerostomia or dry mouth. Xerostomia, or chronic dry mouth, is a common syndrome caused by a lack of saliva that can lead to severe eating difficulties, dental caries, and oral candida infections (Gupta, Epstein, & Sroussi, 2006). In our study was designed to investigate the effects of klotho depletion salivary gland dysfunction on certain aspects of the morphology and cell proliferation rate of mouse tongue tissues. We found that the excessive calcification was observed in the tongue muscle of klotho−/− mice. Therefore, increased elastin fiber in the blood vessel wall and amyloidosis were observed in the tongues of klotho−/− mice compared to klotho+/+ mice. We also demonstrated that cell death induces the tongues in klotho−/− mice and that cell death in tongue may be associated with calcification and fibrosis in muscle and blood vessel wall. These observed klotho may be important to salivary gland function, and may contribute to maintenance of oral health.

In this study, we detected changes in global gene expression patterns in the submandibular glands of wild‐type and klotho‐deficient mouse. This is the first investigation to use genome‐wide screening by cDNA microarray technology to identify changes in gene expression in aged submandibular gland tissue, which consists of mixed cell types such as acinar, ductal, stroma, and fatty, in klotho‐deficient mice.

## CONFLICT OF INTEREST

The authors declare no conflict of interest.
